# Radiologic Aspects of Segmental Odontomaxillary Dysplasia: A Case Report

**DOI:** 10.1155/crid/5963437

**Published:** 2025-11-28

**Authors:** Alessandra Landim, Regina Garcia Dorta, Andresa Borges Soares, Carolina de Paula Rossetto Lisboa, Fabrício Passador-Santos, Alexandre Luís Filócomo, José Luiz Cintra Junqueira, Mariana Quirino Silveira Soares

**Affiliations:** ^1^Oral Radiology Division, São Leopoldo Mandic College, Campinas, São Paulo, Brazil; ^2^Department of Surgery, Stomatology, Pathology, and Radiology, Area of Pathology, Bauru School of Dentistry, University of São Paulo, Bauru, São Paulo, Brazil; ^3^Dr. Paulo Sacramento Hospital and Maternity, Jundiaí, São Paulo, Brazil

**Keywords:** case report, diagnosis, hemimaxillofacial dysplasia, segmental odontomaxillary dysplasia

## Abstract

Segmental odontomaxillary dysplasia (SOD) is a rare nonheritable unilateral developmental disorder characterized by dental, bone, and soft tissue abnormalities. A 13-year-old female patient presented with mild facial asymmetry. Clinical examination revealed right maxillary enlargement and gingival overgrowth. Radiologic examination revealed retention of primary teeth showing pulp chamber obliteration and irregular root resorption, agenesis of the second premolar, impaction of the first premolar, bone enlargement, and increased trabecular bone density with vertically oriented trabeculae. The combined imaging findings facilitated clear differentiation from common imaging mimickers, such as fibrous dysplasia and hemifacial hyperplasia. Bone histopathological examination revealed irregular bony trabeculae lacking an osteoblastic layer, accompanied by numerous basophilic reversal lines. This case underscores the importance of a multidisciplinary approach, combining clinical, radiologic, and histopathological evaluation in distinguishing SOD from other conditions with overlapping radiologic features.

## 1. Introduction

Segmental odontomaxillary dysplasia (SOD), also known as hemimaxillofacial dysplasia, was first described in 1987 by Miles et al. [1]. This anomaly is a unilateral, nonheritable condition characterized by dental, osseous, and soft tissue abnormalities on the same side. SOD was recently included in the World Health Organization (WHO) Classification of Head and Neck Tumors and continues to pose challenges for both healthcare professionals and researchers [2].

Primarily diagnosed in children and adolescents, SOD affects the maxilla, presenting unilateral bone and gingival enlargement, as well as facial asymmetry. Cutaneous manifestations may include erythema, hypertrichosis, and hyperpigmentation [3]. On histopathological examination, nonspecific gingival fibrosis with the absence of inflammation, irregular large bone trabeculae with marked basophilic reversion lines, and the absence of osteoblastic rimming have been reported [4]. Tooth abnormalities are characteristic features of SOD, observed in all reported cases. Unilateral absence of the first and/or second upper premolars occurs in approximately 80% of patients [5]. While clinical evaluation may reveal significant features of the disease, radiologic examination plays a critical role in achieving an accurate differential diagnosis. Imaging modalities are particularly essential to distinguish SOD from conditions such as fibro-osseous dysplasia (FD) [6, 7], juvenile ossifying fibroma (JOF) [8], chronic sclerosing osteomyelitis (CSO), and hemifacial hyperplasia (HH) [5, 6, 9]. Radiographically, SOD is typically characterized by ill-defined dense bone, increased alveolar bone volume, and vertically oriented trabeculae. Bone enlargement frequently results in a reduction in the volume of the adjacent maxillary sinus, further contributing to the distinct radiologic appearance of the disorder [5]. Notably, the WHO identifies radiologic findings as essential diagnostic criteria for SOD [10].

Although approximately 60 cases have been reported [5], the limited literature highlights the rarity of SOD and the critical need for a more comprehensive understanding of its clinical and radiologic features. This case report is aimed at providing radiologists with a detailed description of the clinical, radiological, and histopathological findings associated with SOD, focusing on differentiating it from other imaging mimickers.

## 2. Case Report

**Figure 1:**
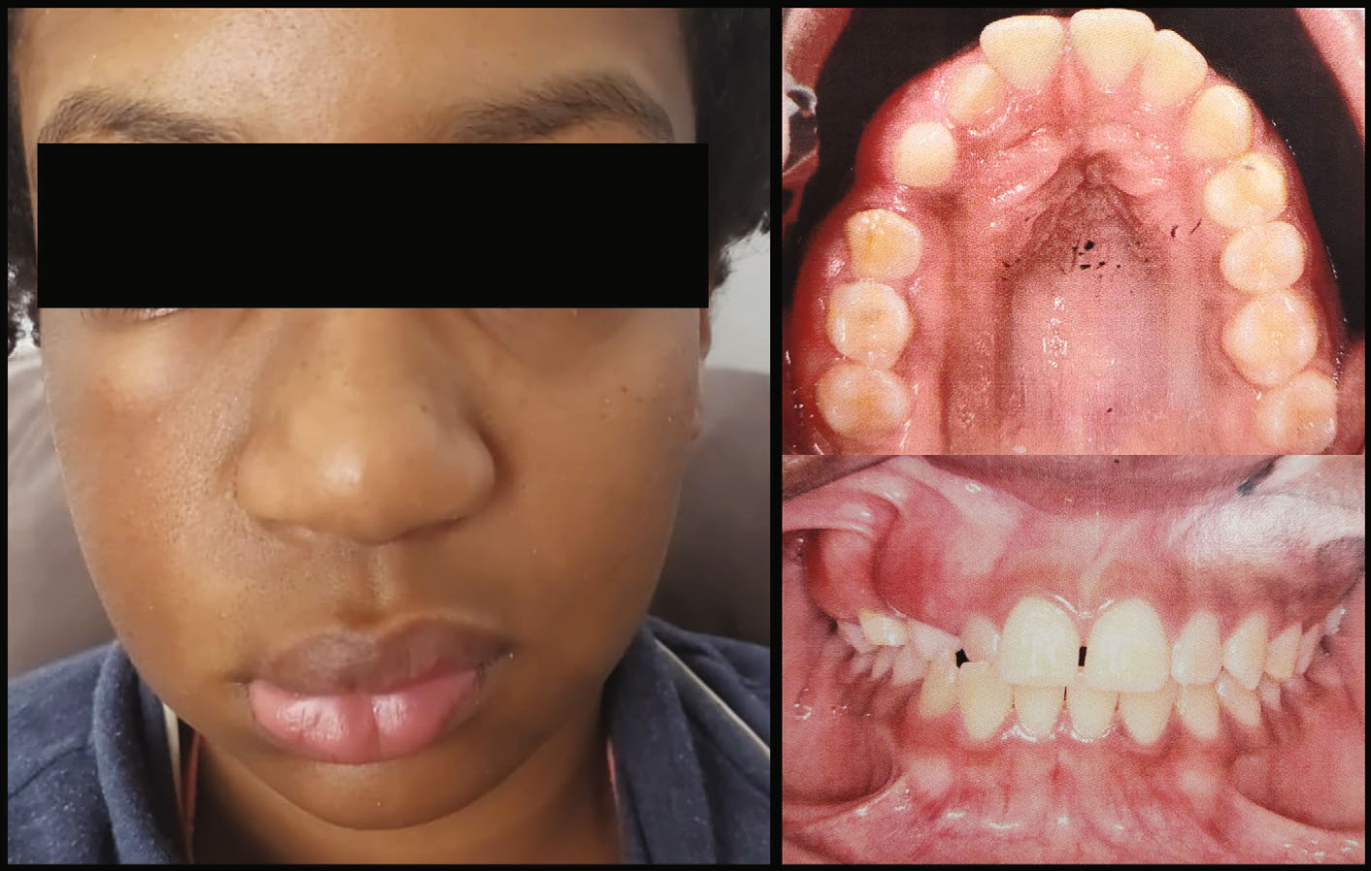
Clinical features in segmental odontomaxillary dysplasia. Facial asymmetry and diffuse right maxillary swelling covered by normal oral mucosa.

**Figure 2:**
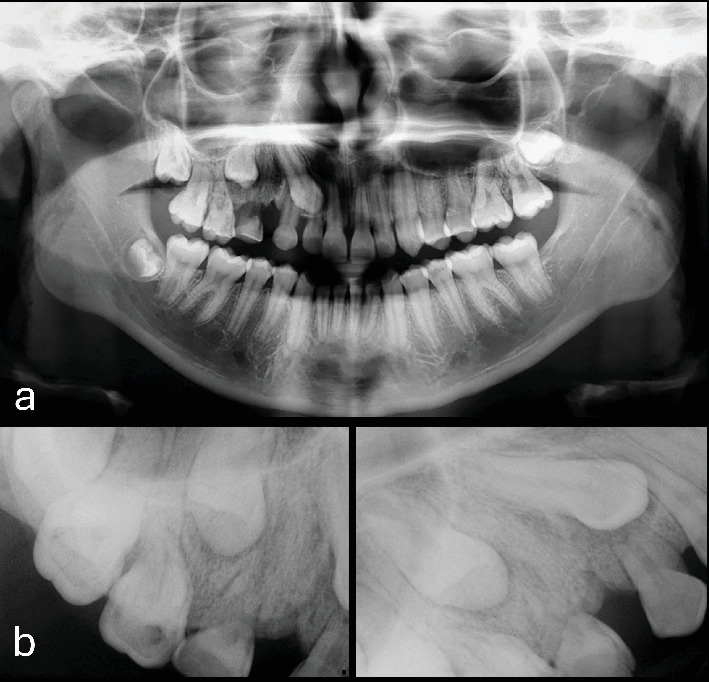
Cropped (a) panoramic and (b) periapical radiographs showing impaction of the right first premolar and canine, absence of the second premolar, and increased bone density with vertical trabecular orientation.

**Figure 3:**
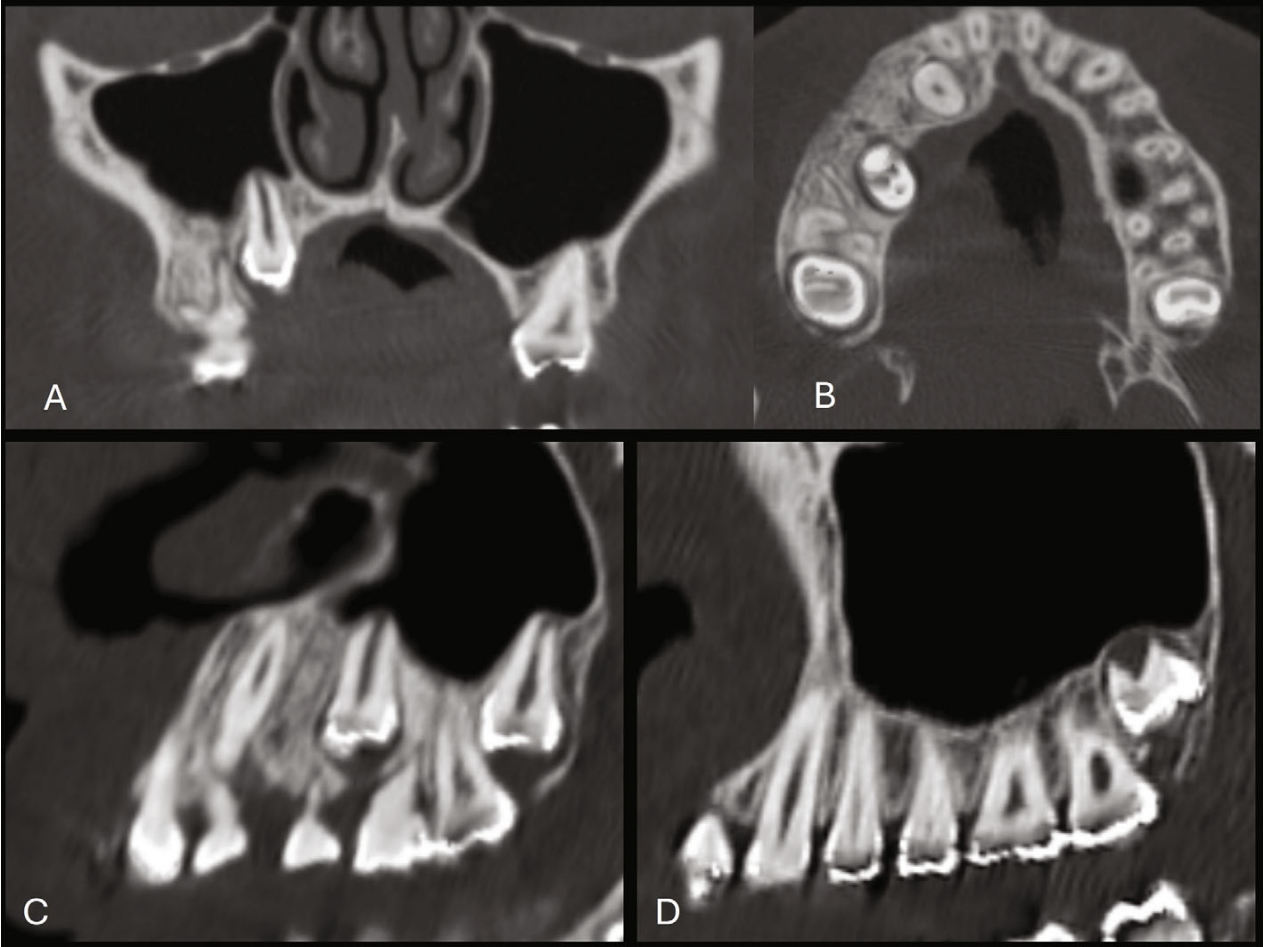
(A–C) Computed tomography reveals increased bone density and bone expansion, accompanied by a reduction in the volume of the right maxillary sinus. (D) In contrast, the left side demonstrates normal tooth eruption, tooth count, bone pattern, and sinus contour.

A 13-year-old female patient was referred to the dental clinic by her parents in November 2022 with the chief complaint of delayed exfoliation of primary teeth and malocclusion affecting the right maxillary quadrant. Her medical history was unremarkable, and her parents denied any systemic diseases or other relevant physical findings. Extraoral examination revealed mild, asymptomatic facial asymmetry. Hyperpigmentation of the upper lip and right midfacial skin was noted. Intraoral examination showed absence of melanin pigmentation in the right maxillary vestibular mucosa, unilateral maxillary enlargement, and localized gingival overgrowth. Panoramic and computed tomography (CT) images demonstrated prolonged retention of the right primary canine and first and second molars, with agenesis of the right second premolar and third molar. The permanent right canine, first premolar, and second molar were impacted. Additionally, CT revealed an ill-defined unilateral increase in trabecular bone density with vertically oriented trabeculae, expansion of the right maxilla, and extension of the bone overgrowth into the maxillary sinus (Figures 1, 2, and 3).

**Figure 4:**
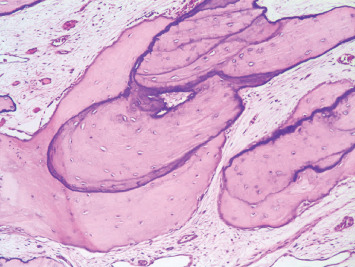
Photomicrograph demonstrating large bone trabeculae exhibiting numerous reversal lines and a lack of osteoblastic and osteoclastic rimming. The stroma is fibromyxoid with the absence of inflammation.

A biopsy of the alveolar bone and gingival tissue was performed. Histopathological examination revealed irregular bone trabeculae, characterized by the absence of an osteoblastic layer and the presence of numerous basophilic reversal lines. The connective tissue displayed variation in density, ranging from dense to myxomatous (Figure 4). Based on the clinical, radiological, and histopathological features, the diagnosis of SOD was established. Genetic testing was not performed due to unavailability at the time of diagnosis.

**Figure 5:**
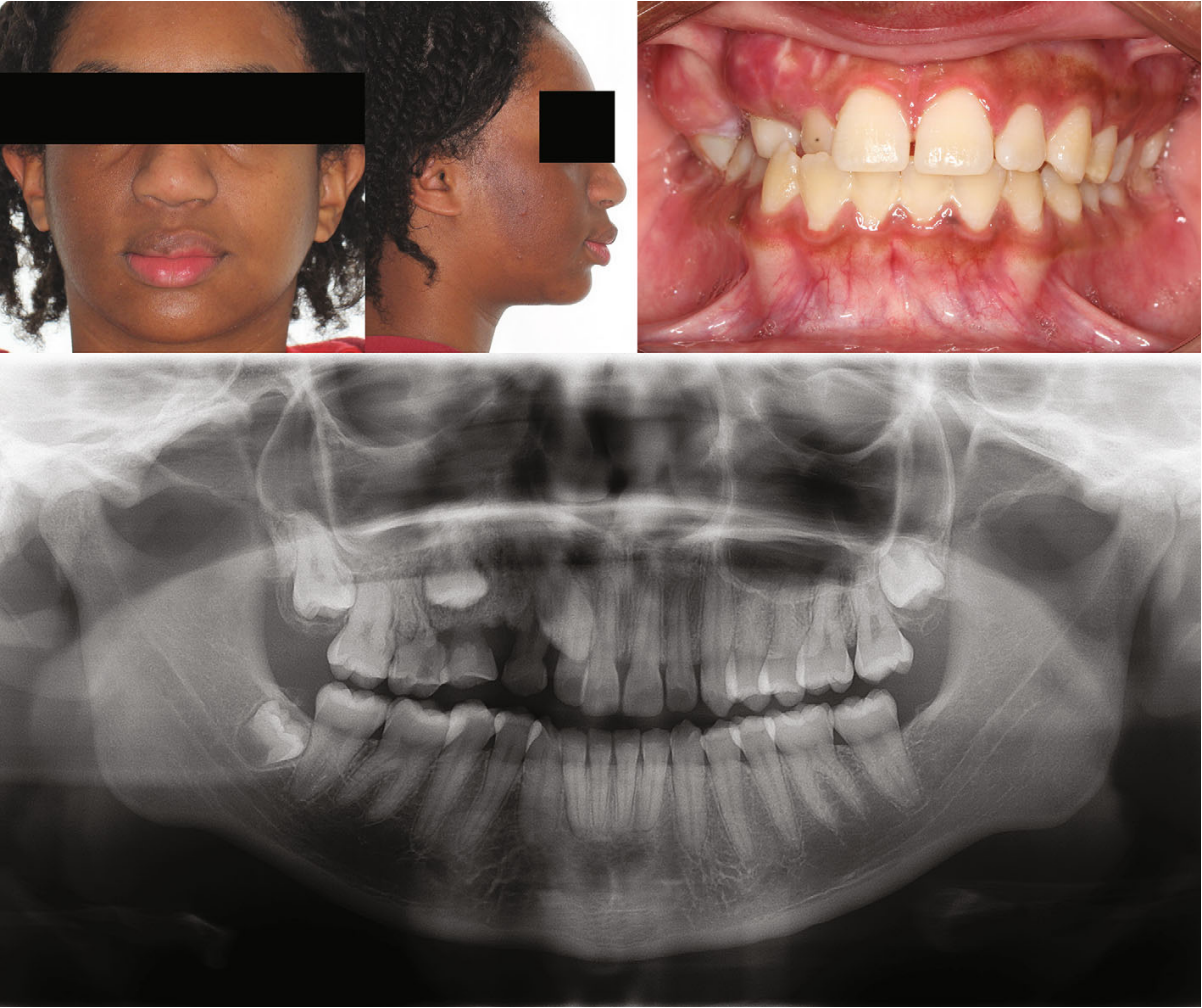
Clinical and radiographic appearance 32 months after the initial diagnosis. No evidence of lesion progression is observed. Clinical photographs highlight facial and alveolar process asymmetry, cutaneous hyperpigmentation of the midface, and hyperpigmentation of the upper lip. The affected side shows absence of gingival melanin pigmentation and prolonged retention of primary teeth. The radiographic image demonstrates findings similar to those at the time of the initial diagnosis.

The patient was referred for orthodontic treatment but was lost to follow-up until July 2025, when she re-presented to initiate care. She was asymptomatic, with no clinical or radiographic evidence of lesion progression. Updated clinical and radiographic examinations were obtained for treatment planning (Figure 5). The interdisciplinary treatment plan included maxillary expansion, extraction of the retained primary teeth, and orthodontic traction of the impacted permanent teeth.

## 3. Discussion

SOD is a rare disorder characterized by asymptomatic unilateral maxillary swelling and facial asymmetry. It predominantly affects individuals around 12 years of age and shows a slight male predominance [5]. To date, isolated case reports and case series remain the primary sources of information about the disease [5, 11]. Although the etiology remains unclear, mutations in the *PIK3CA* or *ACTB* genes have been implicated in SOD [12, 13]. *PIK3CA* is frequently mutated in several types of cancers [13], and notably, several disorders characterized by skeletal and soft tissue overgrowth have been grouped under the term PIK3CA-related overgrowth spectrum (PROS) due to the presence of somatic *PIK3CA* mutations. Some of these conditions share overlapping clinical features with SOD. Additionally, one reported case of mandibular involvement, which is atypical for SOD, demonstrated a *PIK3CA* mutation [13]. Becker nevus is a possible feature of SOD, and it is known that Becker nevus syndrome, which includes some features overlapping with SOD, shares the same somatic *ACTB* mutation. This suggests that patients with both Becker nevus and SOD may share a common genetic alteration involving *ACTB*, thereby supporting the potential value of investigating *ACTB* mutations in addition to *PIK3CA* mutations in such cases [12, 13]. In fact, three patients with SOD were found to carry *ACTB* mutations, as reported by Polubothu et al. in 2018. The WHO emphasizes localized swelling; abnormalities in soft tissues, bone, and teeth; and characteristic radiographic findings as essential diagnostic criteria. Additional desirable criteria include inert bone histologically with defects of tooth formation (only if fibrous dysplasia requires exclusion) [10]. In the present case, both the essential radiologic criteria and the desirable histologic criteria were met, leading to a definitive diagnosis of SOD.

The differential diagnosis of SOD primarily involves monostotic FD and HH. Like SOD, FD represents a painless and progressive bone enlargement during childhood, often diagnosed in the first or second decade. However, unlike SOD, FD solely affects bone, sparing teeth and soft tissues [6]. Radiographically, FD lesions exhibit a “ground glass” appearance, while SOD manifests with vertically oriented thick trabeculae. Histologically, FD displays irregularly shaped trabeculae within fibrous stroma (“alphabet soup” pattern), whereas SOD presents bone trabeculae with numerous basophilic reversal lines within cellular fibrous tissue [4, 6]. Additionally, in SOD, the signs may be present at birth, and in most cases, continuous lesion growth is not observed [5].

Similar to SOD, HH is characterized by unilateral facial asymmetry that may be noticeable at birth. However, HH involves generalized overgrowth of all hard and soft tissues on one side of the face, including the tongue, lips, and bones. This condition is often associated with macrodontia and may exhibit persistent growth into adulthood [9]. While HH presents with uniform overgrowth of the entire viscerocranium [9], SOD is limited to localized changes in the maxilla and associated structures, including sinus volume reduction [5], which helps differentiate the two conditions.

JOF may also present as a unilateral, painless swelling in children and adolescents, often involving the maxillary sinus. Nevertheless, JOF differs from SOD in several key aspects. Clinically, JOF is characterized by a slow-growing, expansile lesion that can displace adjacent teeth, whereas SOD presents with dental abnormalities. Radiographically, JOF typically appears as a well-defined lesion with unilocular or multilocular radiodensity, displaying a mixed radiopaque–radiolucent pattern and well-corticated margins, unlike the poorly defined bone changes, vertical trabeculations, and coarse radiopacities seen in SOD [8].

CSO may also be considered in the differential diagnosis. However, it predominantly affects the mandible and is typically associated with dentoalveolar infections, trauma, or prior dentoalveolar surgical procedures. CSO is commonly accompanied by clinical signs and symptoms, including pain, trismus, fistula formation, and episodes of suppuration [14]. In contrast, SOD has been reported in the mandible in only three cases. All these cases exhibited dental abnormalities, with the homolateral maxillae displaying the characteristic features of SOD [5].

In this case, the patient was referred for orthodontic treatment. While established treatment protocols are lacking, literature suggests a multidisciplinary approach for effective SOD management. Dental implants have yielded successful outcomes in the prosthetic rehabilitation of edentulous areas, demonstrating normal healing and osseointegration. Early diagnosis is crucial for preserving deciduous teeth and facilitating permanent tooth eruption. Definitive treatment is typically postponed until after pubertal growth cessation. Orthodontic treatment may be complicated by soft tissue enlargement, which can interfere with bracket placement and slow tooth movement [5, 15].

## Data Availability

The data that support the findings of this study are available on request from the corresponding author. The data are not publicly available due to privacy or ethical restrictions.
